# Novel Technological Solutions for Assessment, Treatment, and Assistance in Mild Cognitive Impairment

**DOI:** 10.3389/fninf.2019.00058

**Published:** 2019-08-13

**Authors:** Gianmaria Mancioppi, Laura Fiorini, Marco Timpano Sportiello, Filippo Cavallo

**Affiliations:** ^1^The BioRobotics Institute, Scuola Superiore Sant'Anna, Pisa, Italy; ^2^Laboratory of Neuropsychology, USL Nordovest Toscana, Pisa, Italy

**Keywords:** mild cognitive impairment, ICT, cognitive stimulation, neuropsychological measures, cognitive support technologies, social robotics/HRI, dementia-Alzheimer disease, assistive technologies

## Abstract

Alzheimer's disease, and dementia, represent a common cause of disability and one of the most relevant challenges in the health world. In addition, these conditions do not have, at moment, a pharmacological treatment that can stop the pathological progress. Mild cognitive impairment (MCI), defined as the borderline between normal aging and early dementia, represents a meaningful field of study because, in the transition to dementia, clinicians have defined a useful therapeutic window. Additionally, due to the lack of effective pharmacological interventions, recent years have seen an increase in research into new technological solutions to assess, stimulate, and assist patients afflicted with Alzheimer's disease. This review aims to outline the use of information and communication technologies in the field studying MCI. Particularly, the goal is to depict the framework and describe the most worthwhile research efforts, in order to display the current technologies available, describe the research objectives, and delineate prospective future researches. Regarding data sources, the research was conducted within three databases, PubMed Central, Web of Science, and Scopus, between January 2009 and December 2017. A total of 646 articles were found in the initial search. Accurate definition of the exclusion criteria and selection strategy allowed identification of the most relevant papers to use for the study. Finally, 56 papers were fully evaluated and included in this review. Three major clinical application areas have been portrayed, namely “Cognitive Assessment,” “Treatment,” and “Assistance.” These have been combined with three main technological solutions, specifically “Sensors,” “Personal Devices,” and “Robots.” Furthermore, the study of the publications time series illustrates a steadily increasing trend, characterized by the enrollment of small groups of subjects, and particularly oriented to the subjects assistance using robots companion. In conclusion, despite the new technological solutions for people with MCI have received much interest, particularly regarding robots for assistance, nowadays it still owns vast room for improvement.

## 1. Introduction

Worldwide, dementia represents one of the most important causes of disability and reduced autonomy in the elderly population. It is defined, in fact, as a syndrome, generally of a chronic or progressive nature, in which is observed deterioration in cognitive function. Alzheimer's Disease (AD) is the most common type of dementia, with a prevalence of 60–65% of the cases (Alzheimer Association, 2017). According to the statistics and the forecasts about AD, today people afflicted by this condition number approximately 46.8 million. This number is expected to steadily increase over the next few years, reaching 74.7 million by 2030 and 100 million by 2050 (Prince et al., 2016). The growth in dementia cases is resulting in an increase in the associated global costs. Particularly, between 2010 and 2015, the expenditure is expected to increase by 35.5%. Furthermore, projections indicate that the expense will reach $1 trillion by the end of 2018 (Prince et al., 2016). Due to the increase in the elderly population, with an increase in the associated costs, the social impacts, and the apparent lack of effective pharmacological treatments, AD and the others types of dementia constitute a dramatic challenge for the public health services. Furthermore, caregivers of patients with dementia have a higher prevalence of mental health disorders, particularly depression and anxiety (Sallim et al., 2015). For this reason, a strong commitment has been made to find ways to exploit and maximize the remaining cognitive resources of patients with initial symptoms of dementia. These patients, in fact, maintain the ability to learn new skills and strategies, and moreover preserve a good awareness (Olazarán et al., 2004; Belleville et al., 2016). Among all the medical labels developed to describe the pre-dementia stage, Mild Cognitive Impairment (MCI) has been recognized as one of the most useful clinical classifications, and it is one that can represent a therapeutic window for early treatment. This nosographic category defines those who are showing the cognitive depletion which is the manifestation of an intermediate stage between healthy aging with slight cognitive changes and dementia, but able to perform the activities of the daily living, and be essentially autonomous (Petersen et al., 2009). For these reasons, in the last decade, a notable amount of research has committed to the identification of signs and symptoms that could be used as reliable predictive markers of the disease. The MCI early identification, and afterward, dementia identification, would allow the implementation of non-pharmacological interventions that may change the natural history of the disorder, slowing down its development (Petersen et al., 2014).

### 1.1. Traditional Intervention on MCI

Cognitive training (CT) is the most widespread and effective type of cognitive stimulation, among those commonly used in MCI treatment. Particularly, CT protocols take into account bottom-up, and modular tasks aimed at the stimulation of selective cognitive functions, such as memory, language, or, for example, attention (Belleville et al., 2016). Due to their flexibility, CTs are reported as one of the most appropriated technique in the field of MCI. In fact, thanks to their adaptability CTs are particularly recommended with such variegate condition as MCI. On the other hand, more modern stimulation protocols refer to a more complex and holistic model of health that considers physical, emotional, and cognitive aspects. That is due to the high prevalence of Behavioral and Psychological Symptoms of Dementia, namely: agitation, aberrant motor behavior, anxiety, depression, irritability, and apathy, in subjects with dementia (90%) and even in subject with MCI (35–80%) (Monastero et al., 2009; Cerejeira et al., 2012). Particularly, in these cases, apart from the use of CT, are generally adopted, among the others, the music therapy, the multi-sensory behavioral therapy, and the occupational activities (de Oliveira et al., 2015; Massimo et al., 2018). Recently, the relationship between physical practice and other health spheres has become a popular topic. It was demonstrated, indeed, that exercise has a positive influence on hippocampal functions. This effect might facilitate the regulation of long-range cortical networks with a good effect on memory and executive functions (Voss et al., 2010; Chirles et al., 2017). On the other hand, a recent research branch is interested in characterizing the effect of cognitive health on physical activity. It seems that preserved cognitive abilities could allow MCI subjects to perform physical tasks better (Montero-Odasso et al., 2012). According to advanced guidelines, non-pharmacological treatment should possess certain characteristics: they should be performed with high frequency and high intensity; they should provide for a combination of cognitive stimulation and physical exercise; the training should be customized according to the bio-psycho-social characteristics of each participant; and the protocols should be designed in a more ecological fashion and be more generalizable (Belleville et al., 2016).

### 1.2. The Role of ICT With MCI Subjects

In this framework, information and communication technologies (ICT) are accumulating much interest, particularly concerning the applications of these devices in the neuropsychological field. ICTs have been used as assessment tools (Charchat-Fichman et al., 2014; García-Casal et al., 2017) and also as instruments for cognitive intervention (Charchat-Fichman et al., 2014; Ballesteros et al., 2015; García-Betances et al., 2017), enhancing, or, at least, maintaining AD patients' cognitive skills. Although ICTs are more related to patients with a severe grade of impairment, they are now playing an important role as assistive technologies and are serving to promote the independence and the autonomy of individuals with MCI as well as healthy elderly people (Eghdam et al., 2012; Teipel et al., 2016). In light of the information presented in the previous paragraph, technology should play a crucial role in assessment, treatment, and monitoring of MCI patients, allowing the combination of cognitive and physical treatments and melding of the stimulation protocols with subjects' daily activities (Maselli et al., 2018; Fiorini et al., 2019). Moreover, these technologies should be able to aid in gathering data about the changes in the subjects' autonomy and in their physical and cognitive abilities, and give feedback to patients themselves and to the stakeholders (Rebok et al., 2007; Belleville et al., 2016). There remains a paucity of technological interventions for caregivers who are living with people with dementia (Zhang et al., 2016). In this perspective, the need to develop technologies that can be used in the patient's home, without the physical presence of the therapist, is crucial. Moreover, these technologies should be embedded in the user daily life and generate a coupled system with the user him/herself, so to produce an enriched environment (Turchetti et al., 2011).

Although this field is rapidly increasing, ICT applications are generally related to dementia. This study aims to review the literature concerning the use of ICT for assessment, cognitive intervention, and assistance of people who are suffering from MCI, provide a comprehensive view of the current state of the art, and highlight current limitations and future perspectives.

## 2. Methods

### 2.1. Search Strategy

An electronic database search was performed for the period from November 2017 until December 2017 using the U.S. National Library of Medicine (PubMed®), Web of Science (ISI®), and Scopus® databases to identify and select articles concerning the clinical applications of ICTs in the neuropsychological field of MCI. Specifically, the search queries included the following terms: [(Information and Communication Technology OR ICT) OR (Internet of Things) OR (Assistive Technology) OR (Cognitive Support Technology OR CST) OR (Robot)] combined with terms to determine outcomes of interest: (Mild Cognitive Impairment OR MCI) AND [(Cognitive Stimulation) OR (Neuropsychological Assessment)].

The terms research was performed regarding titles and/or abstracts. Only original, full-text articles published in English, which addressed the clinical applications of ICTs on MCI, were included in this review. The reference lists of included papers were examined to identify relevant studies which the electronic search might have missed. Duplicated documents were eliminated; thereafter, the abstracts of the papers, retrieved by the electronic search, were examined to identify which deserved a full evaluation. Finally, similar studies published by the same authors were compared to select the most suitable for our purpose. Obtained in the research were 226 references from PubMedCentral®, 285 references from Web of Science®, and 135 references from Scopus®.

During the process, the identified papers were screened and evaluated from three independent reviewers (i.e., the authors). Meetings and discussions were held to resolve disagreements and find solutions.

### 2.2. Selection Criteria

**Figure 1:**
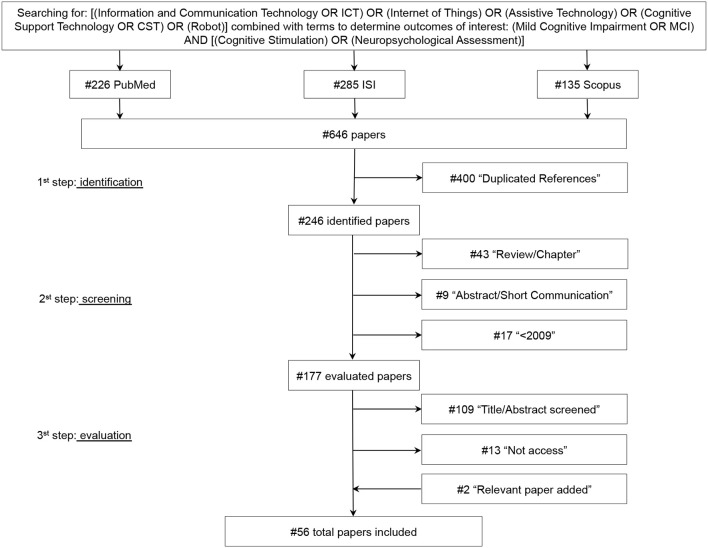
Research methodology for review process.

First, duplicated references were excluded. Then, during the screening procedure, items were excluded if they (i) were an abstract, a short communication, a review article, or a chapter from a book; (ii) were not written in the English language; (iii) were from years prior to 2009. One hundred and seventy-eight references were fully assessed during the evaluation procedure, and papers were excluded if (1) they did not use any type of ICTs; (2) they did not appear appropriate for this review after the reading of title and abstract; (3) they did not address the mild cognitive impairment issue; and (4) they were not full access. In addition, if multiple papers written by the same author had similar content, papers published in journals were selected instead of papers presented at conferences. Furthermore, if multiple papers written by the same author with similar content were presented at conferences, the most recent paper was selected. Finally, 56 papers were fully evaluated and are included in this review (Figure 1).

## 3. Results

Fifty-six papers were selected according to the aforementioned methods and classified on the basis of three major clinical application areas: “Cognitive Assessment,” “Treatment,” “Assistance.”

**Figure 2:**
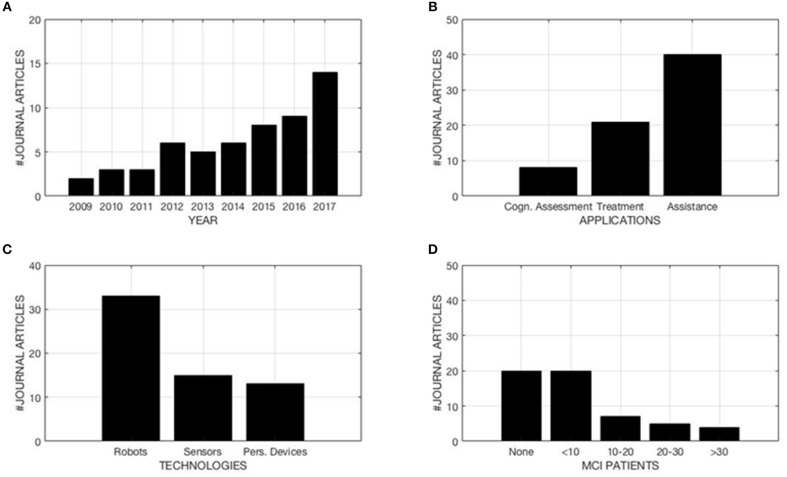
**(A)** Publication trend per year. **(B)** Paper distribution per service. **(C)** Paper distribution per technologies used; Robot (both stand alone and cloud robots), Sensors (both environmental and wearable), Personal Devices (Personal Computer, Smart phones, and TV). **(D)** MCI patients involved in the studies.

Among these applications, the majority of the papers (58%) concern the “Assistance” of subjects with MCI (see Figure 2B). In particular, these articles address the functional assessment, monitoring, and assistance during the daily activities of MCI subjects, in a prosthetic manner. Conversely, the studies labeled as “Treatment” refer to the specific and unspecific cognitive stimulation of MCI subjects, which results in a overall activation, according to Engel's bio-psycho-social model (Engel, 1978). In conclusion, the articles encompassed in the “Cognitive Assessment” category address the use of new technological solutions for the evaluation and the measurement of subjects cognitive performance. They represent, respectively, the 30.4% (Treatment) and the 11.6% (Cognitive Assessment) of the studies reviewed. Note that a certain percentage of the papers reviewed (18.9%) deal with multiple topics (i.e., assistance and evaluation or treatment and assistance) at the same time.

Regarding the technologies used in these works, most of the studies (54.1%) display a scenario in which service robots, both standalone and cloud networked, interact and support people with MCI. The second most represented technologies are sensors, both environmental and wearable, followed by personal devices, including personal computers, smartphones, tablets, and televisions (see Figure 2C). Regarding the sample size of these studies, an ample amount of the research (35.7%) does not include any subject with MCI, but rather healthy controls (HC), caregivers, experts, or no subject at all. This outcome and the general trend represented in Figure 2D can be explained by the theoretical nature of the majority of these studies, or, in a smaller portion, by the primary interest in the technical side. Nevertheless, the growing interest and the technical advances achieved seem to be able to reverse this trend, and lead the scientific community toward more practical implementations, although, nowadays, the clinical validation of the proposed solutions is still a matter of debate. Of the 56 fully evaluated papers, 14 (25%) were published in 2017, while 31 (55.4%) were published over the past 3 years. This result confirms the increasing interest for the ICTs application in subjects with MCI (see Figure 2A).

**Figure 3:**
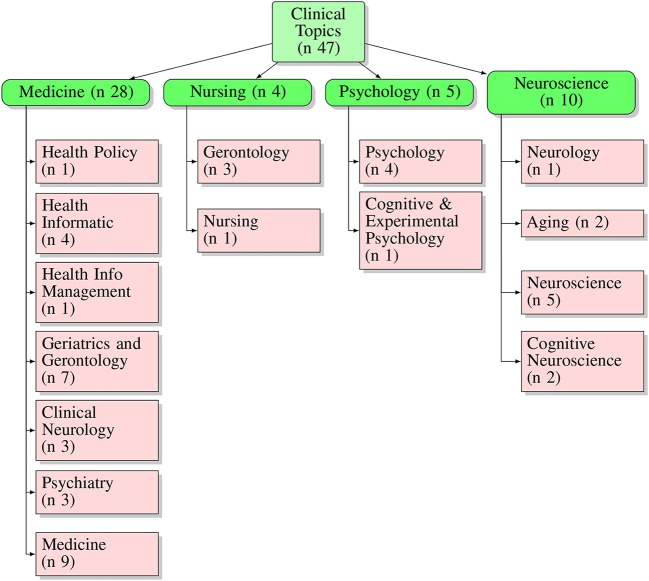
Graphical description of topics touched by articles published in clinical journals. Layer 1, number of general clinical topics; layer 2, main topic categories; layer 3, subcategories of topics.

**Figure 4:**
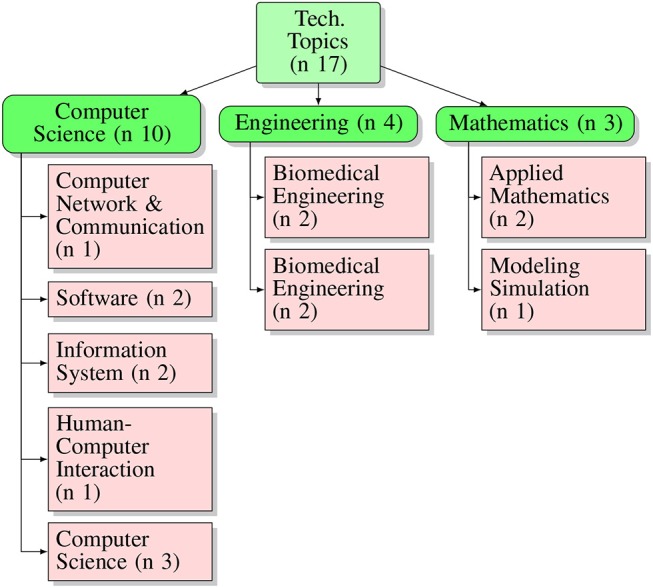
Graphical description of topic touched by articles published in technological journals. Layer 1, number of general technological topics; layer 2, main topics categories; layer 3, subcategories of topics.

Analyzing the journals for the fields of interest of published articles, it can be observed that the leading research sector is the clinical field–17 articles were published in pure clinical journals, indeed. In contrast, only six articles were published in pure technological journals. It is worth mentioning that 4 papers were published in mixed clinical-technological journals. Regarding the clinical side, as summarized in Figure 2A, it can be seen that the leading topic is represented by “Medicine,” particularly concerning general medicine and geriatrics and gerontology topics (see layer 3, Figure 3). The second most sizable category present is, by far, “Neuroscience.” On the other hand, regarding technological journals, it can be seen that “Computer Science” is the technological leading area, followed by the engineering field, equally represented by biomedical and electronic, Figure 4. This analysis suggests a prominent interest coming from the clinician side, with a wider arrangement of objectives, models, and requirements. This interest seems to be not completely matched with the technical community. It could be explained by a high grade of complexity examined by the clinicians, compared with the level of technological advancement. Notwithstanding, the technological community is approaching the topic in a shorter time frame. The average of the publication year for technological journals, in fact, is about 2016. In contrast, the average is about 2014 for clinical journals as well as for mixed clinical-technological journals.

In addition, **Table 2** summarizes the data gathered from the reviewed papers. This table reports some facts, among which are the technology used in the different works, technology service and domain, subjects' clinical profiles, experimental design, and research goal. All 56 papers are illustrated in detail in **Table 2**. In addition, a brief summary of the data included in this paragraph is included in Figure 2 and Table 1.

**Table 1:** Numbers of paper regarding different technologies and their scopes.

	**Cognitive assessment**	**Treatment**	**Assistance**	**Total**
Robots	2	13	26	41
Sensors	3	2	11	16
Personal Devices	3	7	9	19
Total	8	22	46	76

## 4. Application 1: Cognitive Assessment

The category “Assessment” represents the smallest group of papers among all the applications, with only 8 articles reviewed. This category gather articles that aim at the appraisal of cognitive state using both robotic platform, sensors, and personal devices. More specifically, the articles that consider robots are 2, Kintsakis et al. (2015) and Demetriadis et al. (2016); papers that regard sensors and personal devices (in this case PCs) are 6, equally distributed, Dougherty et al. (2010); Zuchella et al. (2014); Manera et al. (2017) use PC, while (König et al., 2015b; Fiorini et al., 2017; Maselli et al., 2017) study the use of sensors.

Below are described the purposes that have pushed the works and the results obtained. A summary of the characteristics of these studies is included in Table 2.

**Table 2:** Papers about evaluation.

**Reference**	**Technology**	**Tech. domains**	**Tech. services**	**Research method**	**Subjects**	**Research goal**
Agrigoroaie and Tapus (2016)	Generic Robot	Assistance	Autonomy	Proof of Concept	None	Present preliminary results from a focus group
Agrigoroaie and Tapus (2017)	Meka M1 Robot Kompaï Robot	Assistance	Emotion	Experimental trial in a HRI scenario	9 HC	Propose a method for extracting and analyzing physiological data
Batista et al. (2015)	Smarphone	Assistance	Social Sphere	Pilot Test in real life	16 MCI	Describe the architecture of the SIMPATIC system as well as its functionality
Bellotto et al. (2017)	Kompaï Robot	Assistance	Autonomy	Lab set test	1 HC	Describe a distributed architecture for AAL services; probabilistic solution for objects localization based on RFID; vision-based approach for estimating the level of activity of a person; entropy-based system for detecting anomalous motion patterns
Broughton et al. (2016)	ENRICHME robot (Kompaï platform)	Assistance	Autonomy	Lab set test	None	Provide the implementation of an library application to detect RFID tags for performing object localization with a mobile robot
Bruno et al. (2013)	Customized Robot Prototype	Assistance	Autonomy Social Sphere Global Cognition	Proof of Concept	None	Define principles and requirements for a wearable SAR aimed at assisting MCI subjects in the execution of everyday activities
Chan and Nejat (2010)	SAR Brian 2.0	Treatment	Memory (LTM; STM) Social Sphere	Proof of Concept laboratory experiments	6 HC	Develop new therapeutic protocols to manage individuals suffering from cognitive impairment by stimulating social and cognitive functioning with a SAR
Darragh et al. (2017)	Generic SAR	Assistance	Autonomy	Questionnaire	7 MCI 2 MD (mild dementia) 8 Caregivers 16 Experts	Gather information about how a robot in the home could assist MCI subjects
Demetriadis et al. (2016)	PROTEAS Tangible Interface for Lego NXT Robot	Cognitive Assessment Treatment	Memory Language Attention Emotion	Test/re-test experiment	25 MCI	Study the usability of a tangible programming interface as a tool for cognitive assessment and evaluate the impact of this type of cognitive training on the patient condition
Diaz-Orueta et al. (2014)	TV Avatar	Assistance	Autonomy Social Sphere	Experimental traial	10 MCI 10 AD	Evaluate what cognitive functions may be involved in the correct interaction with the avatar
Dougherty et al. (2010)	PC-based test battery	Cognitive Assessment	Working Memory Visual spatial Executive processing Verbal Fluency Attention Orientation Processing Speed	Experimental trial	27 MCI 84 AD 104 HC	Compare the accuracy in screening between healthy and cognitive impaired subjects between CST and paper and pencil test
Fiorini et al. (2017)	Inertial Sensor	Cognitive Assessment Treatment	Auditory Sustained Attention	Feasibility Study	4 MCI 11 HC	Present a sensorized approach which combines aerobic exercise and traditional cognitive tools for daily training
Foukarakis et al. (2017)	RAMCIP Robot V1	Assistance	Autonomy	Pilot Study	8 MCI 10 HC	Describe the UI framework, its application in RAMCIP and the initial experiences regarding the use of the framework gathered from the preliminary pilot trials of the project with actual patients
Garcia-Sanjuan et al. (2017)	Customized Tangible-Mediated Robot	Treatment	Working Memory Prespective Memory Episodic Memory Attention Executive Functions	Usability study	12 MCI 12 PWD 16 HC	Present a customized tangible-mediated robot enabling more intuitive and appealing interactions for MCI
Granata et al. (2010)	Robot Kompaï	Assistance	Autonomy	Usability test	5 MCI 6 HC	Study the concomitant use of voice and graphical support to increase the usability of a SAR for MCI support
Granata et al. (2013)	Robot Kompaï	Assistance Treatment	Autonomy	Usability test	11 MCI 11 HC	Present the results from usability testing of grocery shopping list services and an agenda application provided by a SAR for MCI subjects
Gross et al. (2011)	Customized Robot Prototype	Assistance	Autonomy Social Sphere	Proof of Concept	None	
Gross et al. (2012)	Customized Robot Prototype	Assistance	Autonomy Social Sphere	Usability Study Acceptance Study	4 MCI 4 CG	Describes the final implementation of the companion robot and presents results of functional user tests
Kintsakis et al. (2015)	NAO supported by a Cloud System	Cognitive Assessment Treatment	Memory Working Memory Reasoning Awareness	Proof of Concept	None	Present a novel system for performing personalized, robot assisted cognitive exercise and tracking the performance of patients
König et al. (2015a)	RGBD cameras	Assistance	ADL	Lab based test	23 MCI 12 AD 14 HC	Investigate the use of video analyses assessment of IADL
König et al. (2015b)	Audio Technica AT2020 Condenser Microphone Sensors	Cognitive Assessment	Backward Counting Repeating Sentence Describing images Verbal Fluency Task	Experimental trial	23 MCI 26 AD 15HC	Determine the value of automatic analyses of voice recordings during vocal tasks for the early diagnosis of AD
Korchut et al. (2017)	Generic SAR	Assistance Treatment	Global Cognition ADL Emotion Social Sphere	Focus group Surveys	83 MCI 81 CG 100 Experts	Find MCI's needs and preferences toward SAR
Kyriazakos et al. (2017)	Tablet PC Smart-phone Kinect Fitbit Philips Hue Plugwise Pulse Oxi Meter Omron ThinkLabs	Assistance Treatment	Memory ADL	Exploratory study in home environment	48 MCI	Present an opens-source e-Health platform for MCI
Lazarou et al. (2016)	Jawbone UP24 Withings Aura Wireless Tags Plugwise Circles	Assistance	Memory Social Sphere Emotion	Case study	2 MCI 2 AD	Propose a system for continuous and objective remote monitoring of problematic daily living activity areas and design personalized interventions
Mainetti et al. (2016)	Smartphone BLE Beacon GPS Smart Plugs	Assistance	ADL	Proof of Concept	None	Describe the goal of City4Age project
Mainetti et al. (2017)	Accelerometers Gyroscopes Inertial Modules GPS Smartphone BLE Beacon, Smart Appliance (TV)	Assistance	ADL	Proof of Concept	None	Describe the goal of City4Age project
Manera et al. (2017)	Serious Game Virtual Reality	Cognitive Assessment Treatment	Global Cognition	Web-Surveys Workshop	23 Experts	Present recommendations for the use of SGs in assessment and stimulation of MCI subjects
Martínez et al. (2011)	Reed Switch Pressure Sensors Power Plug Sensor Temperature Sensor Smoke Sensor Phone Sensors	Assistance	ADL	Questionnaires Focus group	13 CG 10 Experts	Define MCI behavioral markers
Maselli et al. (2017)	Smart Tissue	Cognitive Assessment Treatment	Episodic Verbal Memory	Feasibility Study	4 MCI 11 HC	Understand system technical viability and its level of sensitivity in measuring memory
Meiland et al. (2014)	Movement Sensors Cameras	Assistance	Memory Social Sphere	Workshops Interviews Expert Consultation	3 MCI 11 PWD 26 CG	Summarize the end users' needs and wishes regarding the development and design of the Rosetta system
Mighali et al. (2017)	Wearable devices Smartphone BLE devices MPU-92509 9-axis MotionTracking device Digital Motion Processor (DMP) ARM Cortex M3 Microcontroller	Assistance	ADL	Lab based test	10 HC	Define a reliable system for controlling the position and the body motility of the elderly in unobtrusive, low-cost and low-power way
Mitseva et al. (2009)	Domestic Sensors Mobile Computer Personal Digital Assistants	Assistance	Autonomy	Proof of concept	None	Describe the initial phases of initiative of offering an intelligent and personalized system for independent living and self-care of seniors with MCI or mild dementia
Muscio et al. (2015)	Serious Game	Treatment	Global Cognition	Proof of concept	None	Define harmonized SGs parameters, and to propose the implementation of biomarkers as enrichment strategy and outcome measures in SGs trial design for MCI
Nakahara et al. (2016)	Customized Robot Prototype	Assistance	ADL	Preliminary study	3HC	Propose a method for logging micro-motion of daily activity based on the skeleton recognition
Nishiura et al. (2014)	PaPeRo Robot	Assistance	Autonomy	Report Case	1 MCI	Reveal how the robot should talk to an older woman with dementia to convince her to perform daily activities
Pahl and Varadarajan (2015)	SCITOS G5 Robot	Assistance	Social Sphere	Proof of Concept	None	Study the use of acoustic sensors utilized for detecting affective haptic inputs
Pino et al. (2012)	Kompaï equipped with a tablet PC	Assistance	ADL	Usability test	11 MCI 11 HC	GUI test
Pino et al. (2015)	RobuLAB10 equipped with a tablet PC	Assistance	ADL Social Sphere	Questionnaire Focus Groups	10 MCI 8 HC 7CG	Investigate SAR acceptance
Reppou et al. (2016)	NAO supported by a smart environment	Assistance Treatment	Attention Memory Awareness Social Sphere	Focus Groups Interviews	6 HC 10 Experts	Describe an architecture system
Sacco et al. (2012)	Cameras	Assistance	ADL	Observational study	19 MCI 16 AD 29 HC	Propose DAS score that detects functional impairment using ICTs in AD and MCI compared with healthy subjects
Schroeter et al. (2013)	SCITOS G3 Robot	Assistance	Autonomy	Interview Questionnaire Observation Robots testing	2 MCI 4 PWD 5 CG	Analyze the added value of a mobile robot companion in a smart home environment, and to evaluate users experience, proving that the robot can act autonomously to provide useful and enjoyable services
Seelye et al. (2012)	VGo Robot System	Assistance	Autonomy Social Sphere	Pilot Study	1 MCI 7 HC	Test the feasibility of use and acceptance of the VGo Robot system
Segkouli et al. (2015)	PC Smart-phone PDA Tablet	Treatment	Memory Attention Judgment Communication Ability	Simulation trial	10 MCI 26 HC	Introduce novel virtual user models with enhanced predictive validity in mental processes that will be utilized for accurate simulation results in interface design
Stavropoulos et al. (2017)	RGB-D Cameras	Assistance	Autonomy	Preliminary study	15 HC	Propose a novel computer vision-based automatic action recognition to increase robustness in realistic assistive robot applications
Tapus et al. (2009)	Custom-designed humanoid torso mounted on a ActivMedia Pioneer 2DX	Treatment	Attention	Pilot Study	2 MCI 7 AD	Develop methods toward SAR therapist for individuals suffering from cognitive impairments through the use of music-based cognitive games
Tiberio et al. (2012)	Giraff Robot	Treatment	Memory Language Emotion	Wizard of Oz experiment	8 MCI 9 HC	Describes a study related to the use of such robots in the interaction with elderly people affected by MCI
Tsardoulias et al. (2017)	NAO robot	Assistance	Attention Memory Autonomy	Focus Group	8 MCI	Propose a novel integrated robotics architecture targeting the needs of individual with MCI at risk for social exclusion
Vasileiadis et al. (2016)	PIR Sensors, RGB-D cameras	Assistance	ADL	Pilot Study	4 MCI	Evaluate a proposed infrastructure for investigating activity monitoring needs
Wu et al. (2011)	Generic SAR	Assistance Treatment	Memory ADL	Interview Questionnaire	30 MCI	Find MCI's needs and preferences toward SAR
Wu et al. (2012)	Generic SAR	Assistance	Autonomy Social Sphere	Focus group	7 MCI 8 HC	Give recommendations about the design of the robot appearance.
Wu et al. (2013)	Nabaztag Robot PC Virtual Agent	Assistance Treatment	Global Cognition Autonomy Social Sphere	Mixed-method: qualitative and experimental	15 MCI 43 HC	Examine the perception of the robot's expression and the role of agent embodiment
Wu et al. (2014)	Kompai Robot	Assistance	Autonomy Social Sphere	Acceptance study	6 MCI 5 HC	Investigate acceptance of a SAR and the effect of direct experience with it over a 1-month period on its acceptance
Wu et al. (2016)	Kompai Robot	Assistance Treatment	Global Cognition Autonomy Social Sphere	Acceptance Usability study	20 MCI	Explore perceived difficulties and needs of older adults with mild cognitive impairment (MCI) and their attitudes toward a SAR to develop appropriate robot functionality
Yamaguchi et al. (2014)	Bono-01 Robot	Treatment	Social Sphere	Experimental trial	10 HC	Propose a robot that warms up group conversations in which have been used conversation technique called “coimagination”, for preventing mild cognitive impairments
Zaccarelli et al. (2013)	SOCIABLE computer battery	Treatment	Reasoning Memory Praxis Executive Functions Attention Social sphere	Efficacy study	106 MCI 118 AD 124 HC	Evaluate the effects of SOCIABLE on the cognition and social sphere, and the affection and the functional abilities of cognitively intact elderly, patients with MCI and patients with mild AD
Zuchella et al. (2014)	Serious Game Virtual Reality	Cognitive Assessment Treatment	Working Memory Prospective Memory LTM Spatial Orientation Selective Attention Executive Functions	Usability test	50 HC	Describe the process used to create the Smart Aging platform for the early identification and characterization of MCI

### 4.1. PC Based Cognitive Assessment

One of the first contributions to the use of PC-based tests for MCI cognitive assessment is the work of Dougherty et al. The authors calculated correlations between subjects' performance in traditional cognitive tests (MMSE and Mini-Cong) and a new PC-based neuropsychological battery called CST. The study results indicate that the CST is a valid and sensitive instrument for evaluating cognitive deficits; in fact, its accuracy in distinguishing between controls and MCI subjects achieved 96%, while the Mini-Mental Status Examination (MMSE) accurately classified 71% and the Mini-Cog 69%. The authors state that PC-based cognitive screening tools may aid in MCI early detection in the primary care setting, and, moreover, due to their ease of use and interpretation, may provide an accurate baseline from which to monitor cognitive changes over time (Dougherty et al., 2010).

Another article about computer-based assessment for people with MCI is the work by Zucchella et al. In this paper, the development and the usability test of a 3D Serious Game (SG), using a virtual environment-based platform for the early identification and characterization of MCI, is described. This SG can record various parameters related to the subjects' cognitive status, including number of correct actions, number of errors, number of false recognitions, number of omissions, and time needed to complete the task. Although the authors claim that SGs could be used in the health domain, in particular in the assessment and rehabilitation of psychiatric and neuropsychological conditions, this usability test underlines problems related to the high complexity of some tasks. For this reason, especially with older people who have limited familiarity with technologies, will need some assistance in the beginning phase. Notwithstanding, SGs have the potential to be new and effective tools in the management and treatment of cognitive impairments (Zuchella et al., 2014).

According to Zucchella et al., a recent work by Manera et al. proposes recommendations for the use of SGs with patients with MCI. Results obtained report that SGs were rated between very adapted and completely adapted for people with MCI. Moreover, SGs are considered as more adapted to people with initial cognitive decline than to people who are already losing autonomy in the activities of daily living. Concerning the use of SGs, participants reported that they found them to range between very adapted and completely adapted for cognitive assessment, as well as to train for physical and cognitive functions, improve well-being, and teach contents. Moreover, concerning the possibility to improve autonomy and social exchanges, they were considered between adapted and very adapted. Importantly, in this paper, the authors stress that the target of SGs, their frequency of use, and the context in which they are played depend on the typology of the SGs (e.g., Exergame, cognitive game), and should be personalized with the help of a clinician (Manera et al., 2017).

### 4.2. Sensors Based Cognitive Assessment

Different types of sensors have been used for MCI cognitive assessment. One example is the work by König et al., in which the authors aim to identify vocal markers correlated to subjects' cognitive status. The classifier developed by the authors showed an accuracy of 79% in discerning between HCs and people with MCI, of 87% in discerning between HCs and people with AD, and of 80% in discerning between people with MCI and people with AD, demonstrating its assessment utility (König et al., 2015b).

Another work was presented by Fiorini et al. The authors designed, and developed, a new tool called SmartWalk System, which aims to assess the sustained auditory attention while the subject walks and simultaneously stimulate the sustained attention domain. The authors compared a traditional test for auditory sustained attention with their SmartWalk system, and the results suggest a good correlation between the two approaches. The results, in fact, show a high significant (*p* < 0.05) correlation for the “correct” and “omitted” scores of the two protocols (*r* = 0.73 and *r* = 0.59, respectively). Also, the “mean” and the “median” response times are significant correlated (*r* = 0.59 and *r* = 0.70, respectively). The authors state that a future research should be focused to increase the number of participants to corroborate the study. Furthermore, a usability study should be planned to estimate whether the SmartWalk system could be used in a daily cognitive training at users' homes (Fiorini et al., 2017).

Similarly, Maselli et al. evaluated the sensitivity of their SmartTapestry System to assess the episodic verbal memory. The results reported by the authors suggest a good correlation between the two approaches. Such findings indicated that the new system was substantially equivalent to the traditional test for the assessment of episodic memory. Furthermore, the results showed a better performance in the consolidation-retrieval process when assessed using SmartTapestry rather than the traditional test. These results suggest that a facilitation in the memory performance may be due to the multiple nature of the mnemonic trace; in fact, the SmartTapestry task involves auditory (the auditory track repeating the list of words), visual-spatial (the position of the letters in the tapestry), and kinesthetic information (the movements of the arms needed to press the letters in the tapestry) that may help the consolidation-retrieval process (Maselli et al., 2017).

### 4.3. Robot Based Cognitive Assessment

Concerning the use of robots in MCI cognitive assessment, it may seem that the development of a robotic assistant, able to assess patients autonomously, would be just a prospect for the future. Nevertheless, Kintsakis et al. proposed the design of a cloud-based NAO robot that aims to engage subjects suffering from MCI during the cognitive test administration. The authors state that use of the robot would increase the compliance and arouse the interest of the subjects during the administration of tests to work memory, arithmetic skills, reasoning, recall, and awareness. Notwithstanding, in this paper, the robotic platform only was presented; the system, in fact, has not yet been evaluated in a real-world scenario (Kintsakis et al., 2015).

### 4.4. Recommendations and Trends

The research for technological solutions able to address the cognitive assessment of subjects with MCI is a fast-growing niche, as demonstrated by the high number of papers (62.5%) in the field that have been published in the past two years. Although the number of articles encompassed in this section does not permit drawing of precise trends, it is possible to observe that some tendencies are emerging in the three different groups of papers. For example, among the articles that examined PC-based technology, it can be seen that the last articles focused their attention more on SGs and virtual reality (VR) than on self-administered batteries of tests, perhaps because they assessed the subjects' performances in a more ecological way (Zuchella et al., 2014; Manera et al., 2017). In a similar way, the use of sensors aims at the detection of parameters that can provide the clinicians with more direct information about subjects' cognitive status, using these parameters as a window on brain activity and functioning. Through the use of a smart environment, it could be possible to detect information about the patients while they perform their activities of daily living (König et al., 2015b; Fiorini et al., 2017; Maselli et al., 2017). In contrast, the introduction of a robotic therapist, able to conduct a cognitive assessment autonomously, which can increase subjects' compliance and arouse their interest, compared to PC-based technology, has, until now, seemed only to be a future prospect and a target to hit (Kintsakis et al., 2015).

## 5. Application 2: Treatment

This category, with 21 articles, is the second most sizable category in this review. Robots represent the technology used most heavily for MCI treatment, with 13 out of 21 articles (Tapus et al., 2009; Chan and Nejat, 2010; Wu et al., 2011, 2013, 2016; Tiberio et al., 2012; Granata et al., 2013; Yamaguchi et al., 2014; Kintsakis et al., 2015; Demetriadis et al., 2016; Reppou et al., 2016; Garcia-Sanjuan et al., 2017; Korchut et al., 2017). On the other hand, papers that consider personal devices and smart environment-based treatment make up 8 out 21: (Zaccarelli et al., 2013; Zuchella et al., 2014; Muscio et al., 2015; Segkouli et al., 2015; Fiorini et al., 2017; Kyriazakos et al., 2017; Manera et al., 2017; Maselli et al., 2017). The articles, organized on the basis of different types of technologies, are presented in the following sections.

### 5.1. Robot Based Treatment

Although robots represent the most commonly used technology in MCI non-pharmacological treatment, generally the purpose of this technology is mixed. Only 5 papers, in fact, address selectively this topic, the others (8 papers) concern also the assistance (6 out 8 papers) and the assessment (2 out 8 papers) of MCI subjects.

The first work related to the use of robots and cognitive stimulation is an interesting paper by Tapus et al., which aims to assess differences in preferences between the use of a robot therapist, instead of an avatar therapist. Basically, with this study they tried to quantify the “embodiment effect” related to the submission of cognitive games through a robot therapist, involving subjects in an 8 month-long stimulation trial. The authors state that the service robot was able to improve or maintain the cognitive attention of the patients with dementia and/or cognitive impairments through its encouragements in a specific music-based cognitive game. Moreover, the robot's ability to adapt maximized the positive effect of the intervention (Tapus et al., 2009).

Chan and Nejat, similarly, proposed a work that aimed at the possibility to develop a robotic therapist that could stimulate and encourage MCI subjects during memory stimulation games. The authors reported a success rate in identifying and recognizing memory game cards between 96%. Moreover, the robot was successful at selecting and executing appropriate emotion-based behaviors throughout the interactions with the participants. Success rate in providing correct behavioral feedback was about 74%. Concerning the participants feedback, 83% of them stated that the robot helped them stay engaged and interested in the memory game (Chan and Nejat, 2010).

As mentioned, emotion recognition and, more generally, the effective channel could provide the robot with useful information for it to model its behavior. A work proposed by Tiberio et al. describes the assessment of tolerance indicators, heath rate (HR), and hearth rate variability (HRV) in subjects who were collaborating with a robot therapist during a cognitive stimulation task. The authors did not find differences between sympathetic activation during the tasks conducted by human or robot. These results emphasize the sample tendency to tolerate the presence of the robot (Tiberio et al., 2012).

Similarly, Yamaguchi et al. presented a robot designed to warm up group conversations of older adults by a cognitive stimulation technique called the “coimagination” method, which is used to prevent the development of MCI and dementia. The authors state that the robot successfully elicited more laughter, which is seen as an enjoyment parameter, than did the human participants (Yamaguchi et al., 2014).

Among the articles about robotic therapists, two, more recent papers, propose an alternative way to administer cognitive stimulation through the robots. Demetriadis et al. and Garcia-Sanjuan et al., in fact, presented two works in which the robot is not an agent that leads people in the cognitive stimulation task performance, but, instead, the robot is the tool for the cognitive stimulation process. In the first case, in fact, the stimulation protocol provides for the use of a programmable tangible interface. So, the stimulation program is made possible through the use of the robot and not the robot assistance (Demetriadis et al., 2016).

In the second example, instead, each participant was involved in the therapeutic use of the robot. Particularly, each participant was asked to perform three different tasks using a tangible-mediated robot: control the orientation of the robot, move the Tangibot from one location to another, and perform a combination of the first two (Garcia-Sanjuan et al., 2017). Contrary to the use of therapeutic robots, the use of a tangible robotic interface is recommended more for people with no or mild cognitive impairments. It seems to be too demanding for those with severe cognitive impairments, but up to now this research field has remained mostly unexplored.

### 5.2. Personal Devices and Smart Environment-Based Treatment

Papers regarding the use of personal devices and smart environment for cognitive non-pharmacological treatment are numerically less represented compared with robotic articles. Despite this, their level of development is, in most cases, higher and better defined compared to the idea of robot therapists or a *fortiori* the use of robots as assistance in the activities of daily iliving.

The first article, here reviewed, about a computer-based cognitive battery, is the work conducted by Zaccarelli et al. In this paper, the authors aim to evaluate the impact of the three-month-long SOCIABLE program on the different cognitive skills. The analysis conducted at the end of the program revealed that MCI patients experienced a positive effect in terms of global cognition, memory, and executive functions. A follow-up examination was conducted to establish the duration of the aftereffects. Examination of follow-up results revealed that healthy elderly individuals showed an increase in memory and language abilities after the use of the program. However, subjects' moods showed an opposite trend and became worse after training, probably due to the increase of self consciousness related to the improvement of cognitive functioning. In conclusion, the authors state that SOCIABLE is an effective intervention suitable for patients suffering from MCI and mild AD (Zaccarelli et al., 2013).

On the other hand, Muscio et al. addressed the topic of SGs for cognitive and social stimulation of MCI subjects. The authors claim that, due to the popularity of video games among the baby-boomers, and a *fortiori* among millennials, video games could be easily turned into enjoyable intervention for cognitive stimulation. In their opinion paper, the authors highlighted the importance of defining harmonized SG parameters and proposed the implementation of bio-markers as enrichment strategy and outcome measures in SG trial design (Muscio et al., 2015).

Another work concerning the use of PC-based technologies is the study by Segkouli et al. The authors developed and tested, a virtual user model (VUM) that simulated the performance of a subject with MCI performing a cognitive task. The purpose of the VUM was to identify and deal with common interface accessibility issues that might occur when people with MCI use PC-based tools for cognitive stimulation. The authors proposed a four-step trial. During the first phase, MCIs and controls were assessed using standard neuropsychological tests and a computer-based battery. Then, during the second phase, the VUM proposed was trained using real users' performances. In the third phase, the authors optimized the VUM and the virtual user interface (VUI). In the last phase, the authors assessed again differences between real and virtual MCIs using the optimized VUI. The authors' new system was able to deal with over 90% of all common interface accessibility issues (Segkouli et al., 2015).

The last, and perhaps, more complete work about the MCI cognitive stimulation, through the use of personal devices, is the research conducted by Kyriazakos et al., also mentioned in section 6. The authors presented the e-Health platform for MCI, in which several personal devices and both environmental and wearable sensors are connected together, via a cloud environment. Among their applications, the e-Health system provides for cognitive games, among which are memory games and tests, attention games, and games using executive functions. This system aims at the preservation of cognitive functions in healthy subjects and especially in MCI subjects. Beyond the good effect on cognition, this type of intervention should help MCI subjects to maintain a good quality of life. This research is still in the design phase, and no results have been reported to date (Kyriazakos et al., 2017).

### 5.3. Recommendations and Trends

The literature about robots and cognitive stimulation is growing faster, and it is becoming recognized by the academic world. For example, about 66% of the papers presented during some conferences address the topic. In this context, it is possible to observe how the majority of the literature concerns the development of a robotic therapist (Tapus et al., 2009; Chan and Nejat, 2010; Tiberio et al., 2012; Yamaguchi et al., 2014). Meanwhile, a small niche of papers concerns the use of robots as tools for cognitive stimulation, instead of as instruments that administer and assist the patients during tasks. A couple of articles reviewed here propose programming or steering a robot to use it for stimulation protocols (Demetriadis et al., 2016; Garcia-Sanjuan et al., 2017). Although the purpose, shared by all the articles in this section, is the stimulation, just the work by Tapus et al. provided for a long-lasting intervention. On the other hand, other articles concern a preliminary study about usability and acceptance. Notwithstanding this lack of experimental data, the literature reviewed here confirms that, generally, elderly people and subjects with MCI prefer robot therapists over virtual therapists. However, generally, they prefer the use of a PC instead of a robot. Despite this, research into the topic has not found differences in perceived stress between the use of PC or robot, neither from physiological signals nor questionnaires. Moreover, research has shown that for robot therapists, along with the ability to administer cognitive stimulation tasks, considering also the emotional and the social spheres is crucial. The positive aspects of using a robot therapist, in fact, is largely related to the embodiment effect, but also to the perception of interacting with a smart agent. For this reason, the ability to empathize with the user cannot be ignored.

However, concerning the use of personal devices and smart environments in cognitive stimulation, it can be observed that this field is less addressed with respect to robotic solutions. Nonetheless, as mentioned, their level of development is higher and better defined compared to that for robot therapists. This fact is also pointed out by the substantial presence of literature published in scientific journals. In fact, just one research effort was presented in a conference and is reported as a proceeding. Generally, the studies of such systems, most of which are PC-based battery, are quite sizable, such as in the case of Zaccarelli et al. (2013). In addition, the time span in which the experiments were conducted was more adequate. In fact, the trials, generally, were scheduled over several weeks. Among the different papers reviewed, as noted previously, the use of PC-based batteries for cognitive stimulation represent the central bulk of contributions. Particularly, different aspects of memory, attention and executive functions were addressed. Moreover, mood and self-awareness were also investigated. A significant aspect of these studies provided for the use of serious games and virtual reality environments to exploit the level of engagement and the ecology of the treatments.

## 6. Application 3: Assistance

The category “Assistance” represent the largest group of articles encompassed in this systematic literature review. This category comprises 40 papers, gathering research from 2010 to 2017, and also in this case the overwhelming majority of the study took into consideration robots as the technology used: 26 out 40 papers (Granata et al., 2010, 2013; Gross et al., 2011, 2012; Wu et al., 2011, 2012, 2013, 2014, 2016; Pino et al., 2012, 2015; Seelye et al., 2012; Bruno et al., 2013; Schroeter et al., 2013; Nishiura et al., 2014; Pahl and Varadarajan, 2015; Agrigoroaie and Tapus, 2016, 2017; Broughton et al., 2016; Nakahara et al., 2016; Reppou et al., 2016; Bellotto et al., 2017; Darragh et al., 2017; Foukarakis et al., 2017; Korchut et al., 2017; Tsardoulias et al., 2017). The remaining papers are shared between two types of technology: namely, personal devices and wearable sensors, making up 14 out 40 papers (Mitseva et al., 2009; Martínez et al., 2011; Sacco et al., 2012; Diaz-Orueta et al., 2014; Meiland et al., 2014; Batista et al., 2015; König et al., 2015a; Lazarou et al., 2016; Mainetti et al., 2016, 2017; Vasileiadis et al., 2016; Kyriazakos et al., 2017; Mighali et al., 2017; Stavropoulos et al., 2017).

The papers are summarized in the following sections.

### 6.1. Robot Based Assistance

The robotic application in the assistance of people with MCI represent the majority of the papers reviewed. As already stated, this is a complex research field because people with Alzheimer's disease require care throughout the day, in different environments, and for varied needs. For these reasons, a significant number of the articles about service robots for assistance are completely theoretical, such as a study by Gross et al., in which they tried to identify the most important functionality for a service robot (Gross et al., 2011), or the work by Agrigoroaie and Tapus, in which the authors suggest that providing further information such as personality attributes, cognitive disability level, emotional internal states, and subjects' preferences would be useful for the process of robot behavior modeling (Agrigoroaie and Tapus, 2016). In an article by Pahl and Varadarajan, the authors suggest the use of unconventional channels to convey meaningful information to the robot, such as haptic inputs for a socially/emotionally based interaction between human and robots (Pahl and Varadarajan, 2015). Concerning theoretical study, an atypical work is the research by Bruno et al., in which the authors proposed the design of a wearable context-aware robot able to share information with a person via speech recognition and production (Bruno et al., 2013).

Another sizable group of papers report that the results came from the clinical and technical experience after focus groups, or were gained through the use of questionnaires and interviews. The work of Wu et al. is an example of that. These authors studied MCI subject's preferences toward robot functionality (Wu et al., 2011) and appearance (Wu et al., 2012). Through their studies they extrapolated seminal insight concerning the embodiment effect, and the interaction aspects between human and robot. The authors state that a robot might offer opportunities for interaction among all members of the elder community, and allow the elders to experience the same power, control, and agency as others.

Similarly, Pino et al. confirmed that learning to perform basic actions using a graphical user interface (GUI) is possible for elderly individuals, either cognitively healthy or impaired (Pino et al., 2012), even though the interfaces should be customized on the basis of the subject's preference, cognitive status, and way of thinking, according to Granata et al. (2010) and (Granata et al., 2013) also investigated the assistive robot acceptance among different groups of older adults living in a community. They evaluated robot and user characteristics, potential applications, feelings about technology, ethical issues, and barriers and facilitators for robot adoption. According to Wu et al. (2012), subjects with MCI preferred robots with animal-like designs instead of the machine-like robots that were preferred by HCs. This study showed that participants with MCI and caregivers had more positive perceptions of the usefulness of the robot than HCs. Furthermore, they recognized the potential of robots for supporting health and social care at home (Pino et al., 2015).

Differently, Schroeter et al. studied the acceptance of robots after participants lived in a smart home for two days, continuously. During this period, the subjects had the chance to interact with the robotia and perform activities such as video calls with short interviews, interviews on site, and also free robot usage. At the end of the trial, information was gathered via interviews and questionnaires. As observed also by Gross et al. (2012), at the beginning of several trial sessions, participants were a bit skeptic about the robot and had stereotypical ideas about robots. After being introduced to the robotic system, all of them expressed interest and appreciation and actually started to think about ways in which the robot could better meet their needs. Most of the users described the trials as an enjoyable experience. Moreover, results also showed that robots were perceived more as a pet (with personality) than as a passive device like a PC or TV, increasing subjects' acceptance (Schroeter et al., 2013).

Recently, Reppou et al. described a novel architectural design for robotic platforms and reported that older adults did not worry about robots and found them useful. Moreover, the authors stated that new technologies and service robots could assist older adults with cognitive impairment by informing them, ensuring their safety with hazard detection, and practicing their cognitive skills with games that stimulate attention and memory. According to Pino et al. (2015), robots should also show emotion and feelings. Reppou et al. (2016) also found that the ability to show emotions is a key feature for a successful robot.

These findings are consistent with more recent studies by Wu et al., who compared cognitive stimulation protocols conducted by computer-based tools in one case a virtual therapist in another case, and a robot in a third case. Although statistical analysis did not show a significant difference among the different methodologies, qualitative analysis revealed the participants' preference for the laptop PC, followed by the robot, and then the virtual agent. The authors stated that individuals with MCI preferred the laptop PC condition mainly because it provided less distracting interfaces compared with the task proposed by the other conditions. Furthermore, the robot was preferred to the virtual agent because of its physical presence, according to some studies (Tapus et al., 2009; Wu et al., 2013). In another study, Wu et al. invited people with MCI and HCs to their living lab to interact with a Kompaï robot once a week for 4 weeks. The study results suggest that both groups could learn and remember how to use the robot, but MCI participants might encounter more difficulties. Moreover, the subjects with MCI did not perceive the robot as useful. However, they found it easy to use, amusing, and unthreatening (Wu et al., 2014, 2016).

The most recent works about theoretical design of service robots define more precisely the users' needs and the robot's functionalities. They found that robots should be able to track physical and psychological well-being, and deliver therapeutic intervention, specifically for individuals with MCI. Two key recommendations for developing a robot for robotic daily assistance were identified. First, subjects with MCI need particular help with daily challenges related to memory issues, including confusion or uncertainty, and help filling the time. Second, the robot should monitor different health indices, such as cognitive skills, movement, and mood (Darragh et al., 2017). Today, researchers studying technological solutions for people with MCI take into account not only the assistance side, but also the chance to stimulate these subjects' cognitive repertoire. The idea behind the latest works is that people with MCI should be helped to communicate with friends and family, keeping themselves informed about regional and international news and weather conditions, but also practicing their cognitive skills with games that exercise attention and memory and supporting them in the rehabilitation process following, for example, a hip fracture (Tsardoulias et al., 2017). The social acceptance of robotic assistants was studied by Korchut et al., who saw robots as a novel tool to improve cognitive functions and prevent cognitive decline, and stressed that the socio-emotional interaction represents a key requirements to create sustainable relationships between elderly individuals and robots. This type of interaction will enhance the users' acceptance and encourage the adoption of the assistive robotic system. For this reason, the robots should be able to understand the psychological state of the user and then provide for positive impact (Korchut et al., 2017).

Even though researchers try to find more and more channels to exploit the communication between humans and robots (e.g., GUI, social/emotional-based communication, and so on), human communication is based on spoken language. The importance to address this topic properly is seminal, taking into account older people affected by cognitive impairment. An example is offered by the work of Nishura et al. The authors presented results from a report case study, in which a PaPeRo robot asked the participants to perform some daily activities, including taking medicines, measuring blood pressure, and cleaning up the room in three different ways for each task. Study results showed that the talking pattern changed the subjects' performances of daily activities (Nishiura et al., 2014). Another example is the study by Foukarakis et al. in which the authors reported that, regarding the robot speech synthesis system, the users had difficulties understanding some phrases. This could be attributed to the quality of the voice used, or maybe to the fact that some of the users had hearing impairments or were old enough to have lower than average hearing, but also the speaking rate setting of the voice could be higher than the optimal setting, considering the target user group (Foukarakis et al., 2017). Introducing new technologies to those who have MCI could be problematic under several aspects, both related with the subjects' cognitive condition, and with general old age issues. The authors found that people with MCI had more difficulty with technology than healthy older adults, and they were confused about the robot's purpose and function. For this reason, technology should be introduced to them as early as possible to give them time to become familiar with it, and to increase acceptance and long-term use (Seelye et al., 2012).

While the aforementioned group of papers comprises studies focusing on the subjects' attitudes toward the robots, another branch of research is more interested in the ability of the robot itself to understand and interact with the subjects, as well as with the environment. Nakahara et al., in fact, did not work on a robot's service, but on the robot's functionality. The authors believed that, to enable the robot to help subjects in their daily lives and to identify risky situations, the robot should be able to recognize humans' activities. The results showed that the developed system was able to recognize correctly the action performed with the following accuracy levels: eating 46%; drinking 59%, calling 26%, walking 89%, writing 25%, reading 40%, cleaning 42%, cooking 43%. This type of technology could be useful for the collection of micro-motion data, which can be used to monitor subjects, but also for the early detection of MCI (Nakahara et al., 2016). Another similar work is the paper by Agrigoroaie and Tapus, in which the authors presented an algorithm that should enable the robot to extract and analyze physiological parameters such as respiration rate, blinking rate, and temperature variation across different regions of the face, to monitor and evaluate the users' emotional states. Particularly, among all the signals analyzed, during this experiment, the authors affirmed that the thermal data represent the most precise indicator of the subject's internal state. In fact, an increased temperature in the periorbital region is related to the growth of anxiety level (Agrigoroaie and Tapus, 2017). Works by both Korchut et al. and Agrigoroaie and Tapus address an important topic: namely, the Human-Robot Interaction (HRI). In this field, as mentioned in previous works, it is becoming crucial to study several interaction channels, even the emotional channel, to ensure a natural interaction between the robot and the users (Agrigoroaie and Tapus, 2017; Korchut et al., 2017).

A last sub-field of research, which takes into account the use of a robotic platform for MCI assistance, addresses the topic of how a service robot should interact with the surroundings to exploit its capability to assist older adults with MCI. A couple of articles concerning this argument are reported below.

As reported by Darragh et al., subjects with MCI need help particularly with daily challenges related to memory issues. For this reason, Broughton et al. and Bellotto et al. focused their attention on how the assistive robot could help the user in practical problems, such as finding objects in the patient's house. At the moment, one of the main problems with robots is that they still have difficulty in perceiving and making sense of the world around them. For this reason, the authors proposed an RFID-based technology that can localize objects (Broughton et al., 2016; Bellotto et al., 2017).

### 6.2. Sensors and Personal Device-Based Assistance

This section encompasses articles concerning assistance through the use of new technological solutions. The independent living of older adults is one of the main challenges linked to the aging population, especially those living with MCI and the consequent frailty. This type of patient needs more support in everyday life and needs to be frequently monitored by formal and informal caregivers. The new ICT solutions, among which are sensors and personal devices, are providing a crucial step forward in the assessment and treatment of these subjects.

One of the first works about the assistance of MCI subjects is the study presented by Mitseva et al., in which the authors evaluated the initial phases of the development of an intelligent system for independent living and self-care of MCI subjects. The authors stated that the starting point is represented by the definition of users' needs, and the proposition of smart solutions for them. They contemplated a three-bundle environment in which users themselves, relatives, and caregivers are immersed together (Mitseva et al., 2009). Similarly, Mainetti et al. discussed the use of wearable and environmental sensors to monitor elderly people with MCI. In their first work, the authors described an unobtrusive system that enables clinicians and caregivers, to monitor the MCI subjects by tracking them during indoor and outdoor activities (Mainetti et al., 2016). In addition, after the authors compared their architecture system with others, they concluded that the key point of their system is its ability to automatically recognize behavioral changes in elderly people with an unobtrusive, low-cost, and low-power technique (Mainetti et al., 2017).

Following the Mitseva et al. instructions, Martinez et al. tried to define MCI needs to develop a smart assistance system. The authors opted for focus group work and *ad hoc* questionnaires to define typical symptoms and behaviors or people with MCI. According to focus group results, the authors determined that the main problems correlated with cognitive decline are forgetfulness, reduction of attention, losing items, forgetting medical appointments, repetitive behavior, difficulty in coordination and organization, use of *paspartout* words, lack of interest in things, and changes in personal hygiene (Martínez et al., 2011). In contrast, Meiland et al. asked MCI subjects, people with dementia, and caregivers to rank the proposed functionality of a smart system in relation to their needs. Consistent with the literature, the functionality most often mentioned as relevant and useful by persons with MCI was “help in cases of emergencies.” However, the functionalities most often preferred by caregivers were support with navigation outdoors and the calendar function. However, some functionalities proposed were not considered useful, such as providing an overview of activities that were performed during the day (Meiland et al., 2014).

As for the other services analyzed, also in this case, the use of sensors and personal device applications is greater than the robot applications. Although the overwhelming majority of papers regarding robots and assistance is relegated to discussing designing and development steps, in contrast, a notable part of this paper encompasses concerns about the system testing step. An example is the work of Sacco et al., which, following the target set by Martinez et al., aims at the usability demonstration of a video monitoring system to obtain a quantifiable assessment of instrumental activities of daily living (IADLs) in subjects suffering from MCI. Experimental protocol provides for specific tasks concerning a daily activity scenario (DAS), performed by subjects while they are recorded with cameras. The authors report that a receiver operating characteristic (ROC) analysis, conducted on the results of the DAS score, showed 89% sensitivity and 73% specificity for discriminating MCI from HC participants. These authors developed an algorithm able to recognize and assess the performance of subjects. Moreover, the DAS score provided a pragmatic, ecological, and objective measurement that might improve the prediction of future dementia and help the clinician to lead an early intervention (Sacco et al., 2012).

Similarly, a more recent paper by König et al. examines the use of fixed cameras in the functional assessment of people suffering from MCI. The authors conducted a two-step experiment in which subjects, both controls and MCI, had to perform a set of physical tasks before, and a set of typical IADLs after, the assessment. All the experiment were recorded u a set of sensors. Koönig et al. reported that the activities were correctly and automatically detected, using an algorithm developed by the authors themselves, with a sensitivity of 85.31% and a precision of 75.90%. The authors noted that the proposed method for monitoring and assessing ADL permits the gathering of objective and accurate information about the functional decline of MCI patients. Moreover, the use of such systems could facilitate and support aging-in-place and improve medical care in general for these patients (König et al., 2015a).

The use of cameras represents a mainstream solution for monitoring of MCI at home Vasileiadis et al. used RGB-D cameras to monitor the subjects' performance in ADL. The authors used cameras and infrared sensors in an eight-day-long test. Then, using the SVM technique, the authors were able to recognize the subjects' activities with a precision and recall rate above 80% using only sensor or tracking data, while the precision rate was over 90% through the combination of both data. Additionally, to test the activity detection potential of a sensor-less infrastructure design, an HCRF-based approach was employed, using only the vision-based features described with data sequences extracted from the occupant's movement, body posture, and upper-body geometry, leading to a precision rate of 90.5%. The authors assessed also the acceptance of the infrastructure, and although participants were hesitant to have guests during the experimental protocol, they showed a positive attitude toward the installations of the sensors in their residences (Vasileiadis et al., 2016).

A last, more recent, work that considers action recognition through cameras is the work of Stavropoulos et al. The authors concluded that a key prerequisite for the development of a service robot, which aims to monitor and support MCI patients at home, is the ability to assess the user's behavior during their daily activities. The authors, starting from the EigenJoints descriptor, developed their own action recognition method. More specifically, they proposed novel features that take into account further descriptive information of the user's actions, such as the traveled distance of the joints and how the user's pose evolves in subsequent frames from the reference frame. The obtained results show that the authors' proposed features improve action recognition performance compared to the original EigenJoints method (Stavropoulos et al., 2017).

The assistance of people with MCI goes beyond the use of cameras—other studies address, in fact, different types of technologies, such as smart phones, as in the case of the paper of Battista et al., but also more complex systems, which combine several wearable and environmental sensors with personal devices.

Some examples of that are represented by the work of Lazarou et al., Mighali et al., and Kyriazakos et al. The first paper is the less complex work on the topic. It provides for a set of sensors and personal devices, which encompass bracelets to evaluate movement, cameras, and devices for sleep monitoring, which provide all the necessary tools to clinicians for efficient monitoring of the participants and promote their quality of life via ICTs by focusing on practical aspects of everyday activities (Lazarou et al., 2016).

The papers by Mighali et al. and Kyriazakos et al. propose a similar concept. Both display a two-block structure, a cloud one and a local one (Kyriazakos et al., 2017; Mighali et al., 2017). Particularly, the first structure takes into account the need to recognize and classify typical elderly activities, such as sitting, standing still, lying down, or walking fast/normal. The authors developed a classifier that showed the capacity to correctly recognize the user's body state with an accuracy level equal to 97% (Mighali et al., 2017).

In conclusion, it is worth mentioning also the study by Diaz et al., which aimed to clarify how some cognitive functions might determine the interaction of MCI with technology. The authors found that, first, some functional measures, such as the Barthel ADL index, are related to the expected number of trials needed by a person for the interaction—for example, with an avatar on TV. Second, cognitive measures, especially those related to attention, processing speed, and discrimination between relevant and irrelevant information, can relate to the latency of response that the subjects show when they respond to the avatar. The authors concluded that cognitive and functional measures may help to predict users' expected response to the avatar. Also, these measures may explain how much time that the interaction will take. For these reasons, the authors noted that cognitive and functional measures should be used for guidance to result in a better adaptation of ICTs to elderly people with MCI (Diaz-Orueta et al., 2014).

### 6.3. Recommendations and Trends

The literature concerning the research in service robots for people with MCI is quite large. This fact can be explained by the fact that, in the overwhelming majority of the cases, the final aim of robots developed for elderly people and/or subjects with MCI are the same they both encompass the design and development of robotic companions for the activities of daily life. For that reason, this section encompasses almost all of the papers about robots, here reviewed. Despite this strong interest in the design and development of robots able to help people with MCI during their daily activities, the technological progress is still in the study of individualization of needs, and in developing usability or acceptability tests. Generally speaking, the sample size of this study is limited, and, moreover, the experimental trial period is a brief and not long enough to draw complete conclusions. Notwithstanding, some results can be presented and some recommendation can be offered. For instance, one of the possible future fields of interest should be the attempt to make the robots more flexible and suitable, to better address subjects' needs. Particularly, concerning MCI, also the stakeholders must be considered. In fact, due to the subjects' conditions, they are not aware of some of their needs, and for this reason they do not find it useful that the robot would be able to remember them of their appointments or when to take their medicines (Korchut et al., 2017).

Concerning the aspects of robots, generally elderly people find that small size, in comparison to human-size, is more tolerated. Furthermore, anthropomorphic or life-like features should be carefully designed with the aim to make the interaction with the robot more intuitive, pleasant, and easy (Wu et al., 2011). Research specifically focused on individuals with MCI reveal that they prefer animal-like shapes and that they like the possibility to interact with the robot, not only via speaking, but also in a more socially and emotionally based way (Pino et al., 2015). In addition, elderly people seem to prefer a robot that looks like a familiar object in a home setting. For this reason, robots might offer opportunities for interaction among all members of the elder community, and they should allow the older people with MCI to experience the same power, control, and agency as others (Wu et al., 2012).

A last thought goes to the tendency of people with MCI to be unwilling to accept an assistive robot for use at home. This observation seems to be a sort of watershed between healthy elderly and people with cognitive impairment. Even if neither the first nor the second are totally enthusiastic to the idea of living with a companion robot, healthy subjects seems to have a more positive attitude toward this kind of robots. For this reason, and thinking to maximize the residual ability to learn how to use robots, it is recommended to introduce elderly people, even those with MCI, to the robots as early as possible (Pino et al., 2012; Seelye et al., 2012). A last comment concerning the attitude of elderly people and this type of technology is drawn to the fact that in coming years we will begin to meet elderly individuals who are increasingly confident and more expert with technologies. This expected change in the way that older will behave should not be neglected.

Concerning the use of personal devices and/or sensors, and their differences with robot-based solutions, this branch of research is not as advanced as in the other fields. This perhaps explains why assistance represents the last step that can be achieved concerning elderly people in general and subjects with MCI, specifically. When comparing the act of assisting people with the act of assessing or stimulating them, assistance is a more complex task. Assistance should provide for monitoring of the subjects, giving them practical assistance, and also giving them social assistance; it requires a higher level of development both in terms of knowledge about pathological condition and in terms of level of technological progress. Overall, when comparing robotic assistance with the idea of assisting people using personal devices, we note that the sample size of the recent is slightly wider, and its trial duration is more appropriate. Moreover, generally, people seem to be more inclined to use assistive technology during their daily activities (Batista et al., 2015). Furthermore, the use of sensors, personal devices, and avatar displayed on TV seem to be less obtrusive (especially regarding cameras), but low-cost and low-power modes are crucial for all of the solutions mentioned (Mainetti et al., 2017). Also, with the use of personal devices, the act of gathering data seems to be simpler. Notwithstanding, the research field appears less appealing and less studied.

## 7. Discussion and Conclusions

Dementia, and particularly AD, is one of the principal causes of disability and reduced autonomy among the elderly population (Alzheimer Association, 2017). It represents one of the most crucial challenging issues that the “health world” will face in coming years, in terms of economic and social costs (Prince et al., 2015, 2016). As mentioned previously, this disease could evolve over as long as twenty years before subjects meet the dementia criteria (Belleville et al., 2016). Due to the minor level of impairment presented by subjects with MCI, it constitutes a valuable therapeutic window for cognitive stimulation (Olazarán et al., 2004; Belleville et al., 2016). From this literature review, despite the limitation of having a research window confined until 2017, it is possible to identify some key points, such as the importance of frequent and intensive sessions of training, the positive influence of a tailored treatment, and furthermore, the value of using an interpretative model that embraces biological, psychological, and social aspects together, to maximize the treatment effect-size. The development of new ICT solutions, usable at the patient's home, without the need for the physical presence of a therapist, allows us to combine cognitive treatments with exercise and social activity (Belleville et al., 2016; Chirles et al., 2017). Although the interest in applying ICT in assessment, treatment, and assistance of people with MCI is steadily increasing, its study is generally related to more severe forms of impairment, such as dementia. However, through a careful literature review, we can recognize several types of ICT applications concerning MCI: “Evaluation,” “Treatment,” and “Assistance.” It is relevant to observe that of the different studies reviewed, most of them encompassed more than application field. In fact, even though the different research works focused their attention on a different, related topic, such as monitoring, for instance, rather than cognitive stimulation, the idea underlying the overwhelming majority of the literature *corpus* generally referred to a complex way to think about the final aim of the technology: namely, the assistance of MCI subjects. In fact, even though the specific papers debated on single topics, the final purpose was to develop a modular, redundant, and synergistic system to take care of the subjects' needs and assist them. Having said that, we encourage the reader to imagine the use of these technologies as spread in a sort of *continuum* among early diagnosis, stimulation, assistance, and monitoring. Even though this field of research has been growing faster in recent years (see Figure 2), some substantial limitations are experienced because of the low number of participants in the different studies. Another index, concerning the novelty of the field, is related to the number of contributors that presented during conferences almost half of the total number of papers. Nonetheless, it is possible to report some considerations and to draw some recommendation for future works.

Concerning the “Assessment,” section, it was not possible to find a dominant type of ICT used. However, SGs and VR will likely be used in increasingly greater numbers. Interesting insights can be drawn from the use of wearable sensors, which would allow clinicians to assess patients during their activities of daily life, increasing the ecology of the measurements. Moreover, thanks to the flexibility of these technologies, they could potentially be combined for assessment and stimulation, both physical and cognitive. The development of robotic therapists just for assessment has been, up to now, minimally studied and used. This kind of solution seems that it will provide a better fit for the stimulation of these patients. The literature regarding stimulation, on the other hand, is more sizable. As just noted, for this application, the development of a robotic therapist is more common, and is one of the driving topics covered in research papers. Notwithstanding the interest gathered by this topic, the literature is lacking experimental data; several works reviewed report usability and acceptance tests. However, some steps forward have been taken. One research study on the topic concluded that a robotic therapist should take into account not only the administration of cognitive stimulation tasks, but also be able to interact with the users in a more emotionally/socially manner. Alternatively, regarding the use of personal devices and smart environments in cognitive stimulation, it is possible to observe a more mature field of research, with wider samples and longer-lasting trials. As aforementioned, regarding the Assessment section, SGs and the use of the VR environment are growing faster and gathering significant interest. Finally, concerning the Assistance section, it represents the widest group of papers, and it is mainly composed of work that examines the use of robots for assistance (Cavallo et al., 2011). Apart from the impressive interest that this field is attracting, it seems to be more a target for future development than a feasible reality, at least for now. That is likely due to a couple of limitations: one, the current state of development for companion robots that assist people, compared to the actual amount of help, in terms of quantity and quality, that the people need, and, two, the massive difficulties in gathering reasonable data. These limitations are primarily related to the complex nature of the service that we need to deliver: assistance. In fact, the gap, present in the other two sections, between the use of robots instead of other technological solutions, is reduced here or, even partially erased. That testifies to the inherent difficulty in assisting properly a human-being, especially one with cognitive impairment.

In conclusion, in our opinion, the challenge should be to address, in a systematical way, the act of steadily stepping up the level of intervention, starting from assessment until complete assistance is provided. Now that users' needs are precisely outlined, and the helpful technologies are identified, a modular, suitable system should be developed that addresses the subjects' strengths and weaknesses; features a smart environment and a cloud architecture; and includes more powerful and intelligent robots. The complexity of the intervention reflects, in fact, the complexity of human beings.

## Author Contributions

GM and LF were responsible for paper structure and writing, and synthesizing the information from the papers into text and tables. MTS was the clinical supervisor, responsible for clinical aspects, and contributed to the introduction, methodology definition and search strategies. FC was the scientific supervisor, guarantor for the review, and contributed in methodology definition, paper writing, discussion and conclusion. All authors were involved in papers screening, selection and reading, providing feedback, and approving the final manuscript.

### Conflict of Interest Statement

The authors declare that the research was conducted in the absence of any commercial or financial relationships that could be construed as a potential conflict of interest.
